# Deletion of a Single Lysine Residue at Position 292 of CAMK2A Disrupts Protein Function, Causing Severe Epileptic Encephalopathy and Intellectual Disability

**DOI:** 10.3390/genes14071353

**Published:** 2023-06-27

**Authors:** Carla Lintas, Angelo Facchiano, Alessia Azzarà, Ilaria Cassano, Claudio Tabolacci, Cinzia Galasso, Fiorella Gurrieri

**Affiliations:** 1 Research Unit of Medical Genetics, Department of Medicine, Università Campus Bio-Medico di Roma, Via Alvaro del Portillo 21, 00128 Roma, Italy; 2 Operative Research Unit of Medical Genetics, Fondazione Policlinico Universitario Campus Bio-Medico, Via Alvaro del Portillo 200, 00128 Roma, Italy; 3 CNR-ISA National Research Council, Institute of Food Sciences Istituto di Scienze dell’Alimentazione—Consiglio Nazionale delle Ricerche, Via Roma 64, 83100 Avellino, Italy; 4 Research Coordination and Support Service, Istituto Superiore di Sanità, Viale Regina Elena 299, 00161 Rome, Italy; 5 Child Neurology and Psychiatry Unit, Systems Medicine Department, University of Rome Tor Vergata, Via Montpellier 1, 00133 Rome, Italy

**Keywords:** neurodevelopmental disorders, intellectual disability, epileptic encephalopathy, exome sequencing, *CAMK2A* gene

## Abstract

Background: The use of NGS technology has rapidly increased during the last decade, and many new monogenic neurodevelopmental disorders have emerged. Pathogenic variants in the neuronal *CAMK2A* gene have been recently associated with “intellectual developmental disorder, autosomal dominant 53″ (OMIM#617798), a syndrome characterized by variable clinical manifestations including mild to severe intellectual disability, delayed psychomotor development, delayed or absent speech, delayed walking, seizures, dysmorphic features and behavioral psychiatric manifestations as autism spectrum disorders, aggressive behavior, and hyperactivity. *CAMK2A* (OMIM*114078) encodes for a subunit of the calcium/calmodulin-dependent serine/threonine kinase II (CaMKII), which is predominately expressed in the brain, where it plays critical roles in synaptic plasticity, learning, and memory as well as in neuronal migration. Methods and Results: We hereby describe a thirty-five-year-old woman affected by severe intellectual disability with epileptic encephalopathy. We performed exome sequencing and found a de novo heterozygous variant in the *CAMK2A* gene (NM_171825.2: c.874_876delCTT; p.Lys292del), which was fully correlated with her phenotype. This is the first report of an inframe single amino acid deletion in a patient affected by intellectual developmental disorder autosomal dominant 53. The variant is predicted to affect protein structure and function and interaction with other proteins and hits a crucial functional site. Discussion: We discuss our variant in relation to previously reported variants and with the objective of delineating possible genotype–phenotype correlations.

## 1. Introduction

With the recent development of NGS technologies, genetic diagnosis has dramatically increased, and many new genes associated with neurodevelopmental disorders (NDs) have been discovered. As a consequence, new ND phenotypes have emerged. Therefore, it seems that we are entering a new era, in which the diagnostic path definitely benefits from a genetic work-up, as it can lead to a precise definition of etiology, family counselling, prognosis forecasting, and gene-based stratification, hoping that targeted therapies are eventually developed. In some cases the clinical presentation of a patient affected by a neurodevelopmental disorder (also in terms of physical traits) can drive crucially the genetic testing, which is often a multi-step process. Unfortunately, in most cases the clinical presentation of the patient is not so specific to suggest a particular syndrome and its causative gene. Therefore, pangenomic tests, both quantitative and qualitative, as array-CGH and exome sequencing must be implemented from the beginning. 

Intellectual developmental disorder autosomal dominant 53 (OMIM#617798, https://www.omim.org/ accessed on 5 April 2023) is an autosomal dominant syndrome recently discovered due to de novo pathogenetic variants in the *CAMK2A* gene (OMIM*114078) [1,2]. Affected individuals display variable dysmorphic features as hypotelorism, epicanthal folds, strabismus, downslanting palpebral fissures, and impairments of the nervous system including delayed or absent speech, delayed walking and psychomotor development. Hypotonia si also reported in many patients. Intellectual disability from mild to severe is always reported, whereas seizures occur in many but not all patients. Additional behavioral manifestations include hyperactivity, autism, and aggressive behavior. Pathogenic mutations have also described in the CAMK2B gene [2], another subunit of the calcium/calmodulin dependent protein kinase II (CaMKII). The associated disorder is named Intellectual developmental disorder autosomal dominant 54 (OMIM#617799, https://www.omim.org/ accessed on 22 June 2023). The disorder is highly similar and partially overlaps with the one associated with the CAMK2A gene. Indeed, poor overall growth, face mild and variable dysmorphic features, strabismus, visual impairment, hypotonia and gastrointestinal problems (feeding difficulties, gastroesophageal reflux, constipation) have been reported. Neurologic manifestations are delayed psychomo-tor development, intellectual disability (from mild to severe), poor or absent speech and seizures (in some patients). Pathogenic variants arise de novo as for the CAMK2A gene.

The clinical database *ClinVar* (https://www.ncbi.nlm.nih.gov/clinvar, accessed on 10 March 2023) reports to date (March 2023, Figure 1A) 13 pathogenetic SNVs (single-nucleotide variants) and 13 likely pathogenetic SNVs for the *CAMK2A* gene. Variants are predominately missense (16), followed by stop/frame shift (6), and splicing (3) variants and one inframe deletion of two amino acids (1).

In the database *GnomAD* (https://gnomad.broadinstitute.org/gene/, accessed on 10 March 2023), the *CAMK2A* gene is intolerant to both missense and loss-of-function variants. Biallelic variants in *CAMK2A* have also been recently associated with a similar but more severe intellectual disability syndrome known as intellectual developmental disorder autosomal recessive 63 (OMIM#618095). Chia et al. reported an homozygous missense variant (His477Tyr) in *CAMK2A* in two severely affected siblings [3].

**Figure 1:**
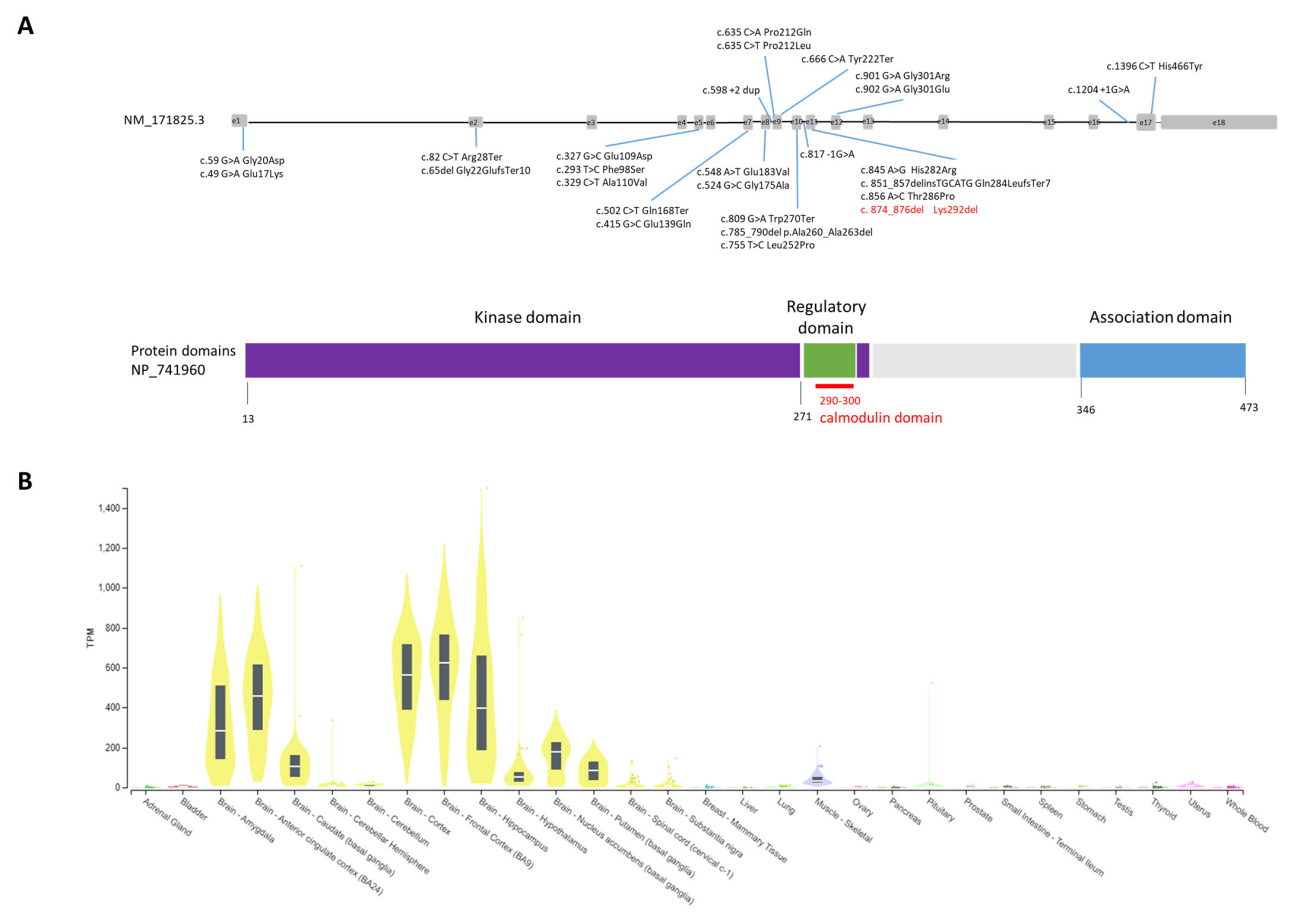
(**A**) *CAMK2A* gene and reported pathogenetic variants to date; gray boxes are exons, whereas introns are black lines not in scale. Domains of the protein are shown in dark blue (kinase domain), green (calmodulin domain) and light blue (association domain). (**B**) Tissue expression profile of *CAMK2A* from GTExPortal (https://www.gtexportal.org/home/gene/CAMK2A) (accessed on 20 June 2023). On the Y-axis, mRNA expression level for CAMK2A is shown by the yellow bars. Expression is high in most brain areas.

*CAMK2A* encodes for a subunit of the calcium/calmodulin-dependent protein kinase II (CaMKII), which is a dodecamer encoded by other three additional genes: *CAMK2B*, *CAMK2G*, and *CAMK2D* [4,5]. *CAMK2A* and *CAMK2B* display high homology and have four distinct domains: a catalytic domain characterized by an active site required for the kinase activity of CaMKII, a regulatory domain containing the calcium–calmodulin binding site, a variable domain that displays low homology between these two genes, and an association domain necessary for the assembly of the twelve CaMKII subunits. CaMKII is critical for synaptic plasticity and for the regulation of synaptic strength through the interaction with ionotropic glutamate receptors such as NMDAR and AMPAR [4,5]. This process is triggered by calcium ions’ entry into the cell, and consequent calcium–calmodulin binding to the regulatory domain causes phosphorylation (threonine 286 for *CAMK2A* and threonine 287 for *CAMK2B*) and activation of the CaMKII protein. Pathogenic heterozygous variants of *CAMK2B* have been described in association with a neurodevelopmental disorder known as intellectual developmental disorder, autosomal dominant 54 (OMIM#617799). For both genes, pathogenetic variants reported to date are located mostly within the protein kinase domain and in the regulatory domain (Figure 1A). Expression of the *CAMK2A* gene (https://gtexportal.org, accessed on 10 March 2023) is brain-specific and is the highest in the cerebral cortex followed by the hippocampus, the amygdala, and the basal ganglia (Figure 1B).

In this report, we describe a thirty-five-year-old woman severely affected by intellectual disability and epilepsy, with a novel mutation in the *CAMK2A* gene. The variant arose de novo and consisted of a single amino acid deletion (NM_171825.2: c.874_876delCTT; p.Lys292del) located within the calcium–calmodulin domain. In addition to the patient description and the variant causing the phenotype, we per-form a deep bioinformatics analysis showing how the p.Lys292del detected variant af-fects negatively its protein structure and its interaction with other proteins. We also search the literature for patients carrying pathogenetic CAMK2A variants with the specific objective of delineating possible genotype-phenotype correlations.

## 2. Materials and Methods

### 2.1. DNA Extraction and Exome Sequencing

Peripheral blood was collected in two EDTA tubes, and genomic DNA was extracted using the QIAamp DNA Blood Mini Kit (Qiagen, Milan, Italy) following the manufacturer’s instructions.

Whole-exome sequencing was done in the trio (proband and her parents), with an average coverage of 60× on an Illumina platform. The bioinformatic analysis was performed on the online platform Galaxy [6]: the fastQ files were aligned using the Burrows–Wheeler Aligner [7] (Human GRCh38/hg19); duplicates were removed using RmDup to perform the variant calling with FreeBayes and the variant annotation with wAnnovar [8].

All bioinformatic analyses were performed following best-practices recommendations [9,10]. We included in our analysis all variants identified within the coding (missense, stop, frameshift, and indels) and the splicing regions with a good coverage. We prioritized de novo variants. We also filter for variants with a population frequency in the *GnomAD* database (https://gnomad.broadinstitute.org/, accessed on 10 March 2023) lower than 1%. The variant was classified according to the American College of Medical Genetics guidelines [11]. In addition to *GnomAD*, many online bioinformatics tools were consulted, including OMIM (http://www.omim.org/, accessed on 10 March 2023), *ClinVar* (https://www.ncbi.nlm.nih.gov/clinvar/, accessed on 10 March 2023), Varsome (https://varsome.com/, accessed on 10 March 2023), Mutation Taster (http://www.mutationtaster.org/, accessed on 10 March 2023), Polyphen-2: (http://genetics.bwh.harvard.edu/pph2/index.shtml, accessed on 10 March 2023), Decipher (https://decipher.sanger.ac.uk/, accessed on 10 March 2021), Ensemble (https://www.ensembl.org/index.html, accessed on 10 March 2021), and SFARI (https://gene.sfari.org/, accessed on 10 March 2021).

### 2.2. Sanger Sequencing

Sanger sequencing was used to validate the potential pathogenetic variant in the proband and in her parents. Primers flanking the variants were designed using Primer3 application on the UCSC genome browser. The 5′-3′ sequence of forward and reverse primers were “agacccaggcagagctagtc” and “agagactgcccctgctgtgg”, respectively. Using manufactures guidelines, PCR products were cleaned up using a mixture of Exonuclease I and Shrimp Alkaline Phosphatase (ArticZymes, Tromsø, Norway), sequenced using BigDye terminator Kit (Applied Biosystems, Foster City, CA, USA), and run on a 3500xl Genetic Analyzer (Applied Biosystems, Foster City, CA, USA). The electropherograms were analysed by the Sequencing Analysis software (Applied Biosystems, Foster City, CA, USA).

### 2.3. Protein Modelling and Structural Analysis

The 3D structure of CAMK2A protein is available in the Protein Data Bank (PDB) by different entries. We used the 3SOA entry as template for the modeling procedure because it has the most coverage of the sequence, i.e., 444 amino acids out of 478, with the second choice being struture 2VZ6, with 313 out 478 amino acids. The modeling procedure was applied as for the previous comparative modeling studies of our laboratory [12,13]. Modeling was performed by the Modeller 10.4 tool [14]. Images of models were created and structural analyses were performed by DiscoveryStudio v.4.5.1, Dassault Systèmes (San Diego, CA, USA).

Further experimental models in PDB of CAMK2A would be interesting for the study of the interaction of CAMK2A with other proteins. PDB entry with code 7B57 reports the structure of mouse CAMK2A complexed with human α-actinin-2, and it was used for analyzing the interaction region. Interface interactions were detected by the PDBePISA tool [15].

## 3. Results

### 3.1. Clinical Description of the Proband

**Figure 2:**
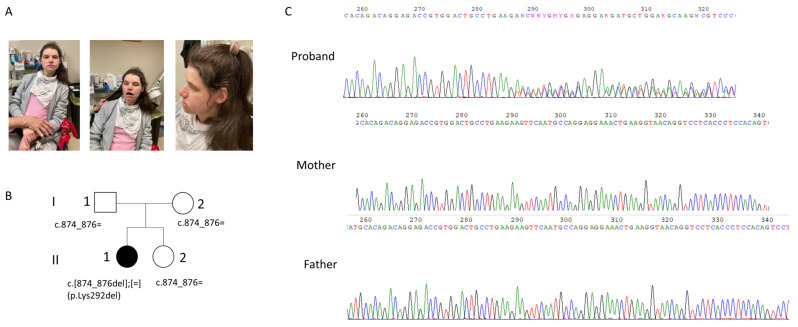
(**A**) Patient; evident dysmorphic features include microcephaly, marked prognatism and thick vermilion borders; additional patient features are reported in the text. (**B**) the genealogical tree of the family; (**C**) electropherogram of our patient and her parents showing the Lys292del in the proband. The Lys292del variant is not present in the mother and in the father as can been seen by the electropherogram.

The patient came to our medical genetics service accompanied by her parents when she was 30 years old for a new genetic evaluation. She had several previous genetic evaluations, which did not helpful to reach the diagnosis. Previous specific genetic tests for Angelman syndrome and Rett syndrome were negative; array CGH analysis reported a maternally inherited duplication of the 15q11.2 region (about 2.2 Mb) and a paternally inherited deletion at the 3q25.1 region (about 170 Kb). Additionally, a multigenic panel for epilepsy (11 genes) did not detect any clinically significant variant. Therefore, exome sequencing was performed. The proband was the first of two siblings (Figure 2A,B) as shown by the genealogical tree of the family.

The clinical history was characterized by severe delayed psychomotor development, with seizures starting at puberty. The child was born by vaginal delivery at a gestational age of 39 weeks with a birthweight of 3.5 Kg and a birth length of 52 cm. (occipito-frontal circumference (OFC) was not reported.) She had no history of intrauterine or perinatal distress, and Apgar score was 9 and 10 at 1 and 5 min. Psychomotor delay became evident in the first year of life, as she showed early hand stereotypies. The OFC was 45 cm (25–50°) at 1 year, then, there was a deceleration in head growth, and the OFC was 47 cm at 4 year (<2 SD). She did not develop language and reached a standing position with support at 4 years. At the time of our clinical evaluation, the patient was nonverbal, unable to walk, and was affected by severe intellectual disability. Hyperactivity and oral and hand stereotypies were noticed. Physical anomalies included microcephaly, marked prognatism, and thick vermilion borders. She was hypotonic.

She had osteoporosis and slight scoliosis. The patient received antiepileptic medication (ASM), including carbamazepine and topiramate, with a good control of seizures.

Her wakefulness EEG reported monomorphic theta rhythm (6–7 h/z) with slow activity in the anterior regions without epileptiform discharges.

### 3.2. Genetic Findings

Trio WES analysis revealed that the proband (Figure 2A) carried a de novo variant in the *CAMK2A* gene NM_171825.2: c.874_876delCTT, p.Lys292del, in heterozygosity. The variant was confirmed by Sanger sequencing (Figure 2C). According to the ACMG (American College of Medical Genetics) guidelines [11], the variant can be classified as pathogenetic for the following criteria: it is a de novo variant (PS2 criteria), it is absent from the *GnomAD* 2.1 population database (PM2 criteria), it is located in a well-established functional domain (PM1 criteria), and the protein length changes as a result of the inframe deletion (PM4 criteria). In addition, the in silico prediction tool “SIFT for indels” (https://sift.bii.a-star.edu.sg/www/SIFT_indels2.html, accessed on 10 March 2023) predicted a “damaging” effect for the p.Lys292del. The PhyloP100 (https://genome.ucsc.edu/, accessed on 10 March 2023) conservation score is very high (score equal to 8.017), indicating a high conserved amino acid position for Lys292 across different species.

### 3.3. Protein Structure

**Figure 3:**
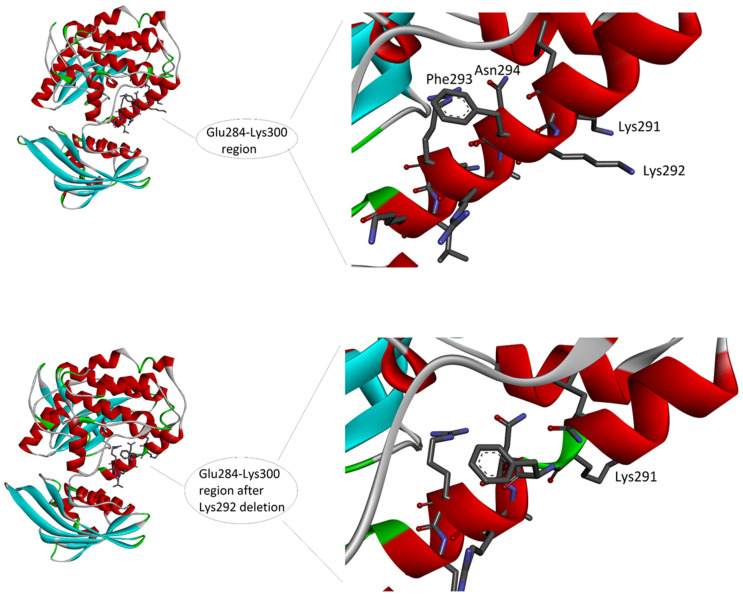
The 3D structure of the complex between CAMK2A protein (green) and alpha-actinin-2 (blue) (PDB code: 7B57). On the left, the whole complex. On the right, the enlarged view of the interacting region, to evidence the involvement of the CAMK2A helix that includes Lys292. Interactions at the protein-protein interface, as detected by PDBePisa tool (see Methods) are listed in the lower part of the image. The interacting atoms are indicated by arrows in the right panel, except for A:Tyr889[OH] and B:Ala302[O], because hidden by the ribbon representation of the protein backbone.

Deletion of Lys292 occurs within the calmodulin binding domain, which covers the 290–300 region of the sequence (according to *UNIPROT* database: https://www.uniprot.org/uniprotkb/Q9UQM7/entry, accessed on 10 March 2023). The sequence of this domain is L(290)KKFNARRKLK(300). The domain consists of eleven amino acids, and six of them are positively charged (i.e., four lysines and two arginines, represented by K and R in the sequence, respectively). Charges are important for the function of this domain, as it is reported that the calmodulin binding domain is typically an amphipathic helix with positive charges (Figure 3).

The amphipathic feature is given by the alternation in the sequence of hydrophilic residues, i.e., the six positively charged residues and an asparagine (N), with hydrophobic residues, i.e., leucine (L), phenylalanine (F), and alanine (A). Due to the helical conformation, which presents on the backbone of the side chains of the amino acids with a step forward of 100 degrees, all the hydrophobic side chains are approximatively oriented on the same side of the helix surface, while the hydrophilic side chains are on the opposite side. The deletion may have a double effect on the helix conformation and function. Firstly, the positive net charge is reduced, with a possible loss of capability to interact with the negative calmodulin. However, this expected effect could not change the binding ability, as reported in literature [16]. Secondly, the deletion induces either a break in the helical conformation of the backbone, thus altering the conformation (Figure 3), either a shift in the helix disposition of amino acid side chain (not shown), with a possible loss of the amphipathic distribution of the side chains. In fact, if amino acids from 293 to 298 (which in the helix follow the deletion) would undergo a rotation, thus changing by 100 degrees the position and exposure of each of them on the surface of the helix, the amphipathic feature may be loss, thus altering the capability of this region to form correct interactions in protein–protein binding.

To investigate the functional role of the region where the deletion occurs, we analyzed the available structures of CAMK2A or very similar proteins in complex with protein interactors. Unfortunately, the other structures of human CAMK2A in PDB do not represent complexes involving the region of interest. However, the mouse CAMK2A complexed with human α-actinin-2 is available in the PDB (entry code: 7B57). Mouse and human CAMK2A are very similar in their sequences, with only one different amino acid in position 324 (i.e., Asn in mouse and Ser in human sequence). Therefore, we can assume that a similar interaction can occur in the case of human CAMK2A.

**Figure 4:**
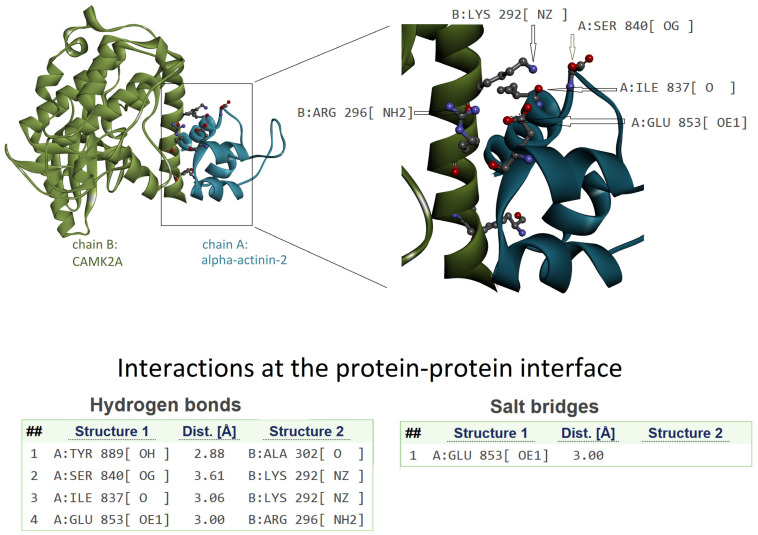
The 3D structure of the complex between CAMK2A protein (green) and α-actinin-2 (blue) (PDB code: 7B57). On the left is the whole complex. On the right is the enlarged view of the interacting region to evidence the involvement of the CAMK2A helix that includes Lys292. Interactions at the protein–protein interface, as detected by PDBePISA tool (see Methods), are listed in the lower part of the image. The interacting atoms are indicated by arrows in the right panel, except for A:Tyr889[OH] and B:Ala302[O] because they are hidden by the ribbon representation of the protein backbone.

The complex structure is shown in Figure 4. The CAMK2A region of interest is directly in contact with α-actinin-2, and Lys292 is involved in two H-bond interactions, as evidenced by the analysis of the interface. Therefore, it is evident that the deletion of Lys292 can reduce the capability of interaction with α-actinin-2. Moreover, a salt bridge interaction involving Arg296 could be disrupted in case of deletion and a change of orientation of the Arg side chain.

## 4. Discussion

In 1992, Silva and colleagues demonstrated that mutant *CamK2a* mice exhibited specific spatial learning impairments, deficit in synaptic plasticity and memory [17,18]. Heterozygous *Camk2a* knock-out mice displayed a decreased fear response and an increase in defensive aggression; at a cellular level, serotonin release was reduced [19]. Later, de novo heterozygous mutations in the *CAMK2A* gene were reported in individuals affected by intellectual disability of variable degrees [1,2,20]. Our patient phenotype perfectly matches with that of other patients carrying *CAMK2A* pathogenic variants; shared phenotypic traits include severe intellectual disability, seizures, microcephaly, absent speech, developmental and motor delay. Some of these traits as seizures or microcephaly, are not always present in all patients, and the degree of severity of their manifestation may be very variable. Microcephaly, for instance, was reported by Kury et al. [2] in one patient out of fourteen and by Akita et al. [20] in one patient out of three carrying *CAMK2A* pathogenic variants. Intellectual disability is the only trait shared by all *CAMK2A* patients. However, no correlation between type of mutations (missense, splicing, or frameshift) and the degree of intellectual disability has been reported (Table 1).

The absence of genotype–phenotype correlations with respect to intellectual disability is confirmed by the observation that even unrelated patients carrying the same Pro212Leu missense mutation (patient 10, 11, and 12 of Table 1) have a different degree of intellectual disability, ranging from mild to severe (Table 1). Similarly, seizures appear not to be correlated with the type of mutation: patients carrying missense variants may have or not seizures, and the same observation is also valid for patients carrying frameshift or splicing variants (Table 1). In general, variants falling within the regulatory domain are associated with a more severe intellectual disability phenotype compared to variants falling in the kinase domain regardless of the mutation type (Table 1). However, additional studies are needed to confirm this association.

In the case of our patient, her severe phenotype (severe intellectual disability, absence of speech, epilepsy, and inability to walk) might be related to the type of variant she carries. Indeed, Lys292 falls within the calmodulin domain, an important functional domain of the protein encoded by the *CAMK2A* gene, suggesting that the loss of this amino acid may have significant biological consequences. Hence, it is likely that the loss of one positive charge could significantly affect the function of this domain, which contains six positive charges (four lysine K and two arginine R) and no negative charge. This has been explained in detail in the Results section (Figure 3). The single amino acid change, i.e., Lys292del, will ultimately affect the possibility for this region to interact with other proteins.

Many pathogenetic variants identified in patients have been functionally characterized by using cellular or in vivo models. Akita and colleagues [20] used neuro-2A neuroblastoma cells and primary hippocampal neurons to model the variants identified in their patients. The Pro212Gln and Pro235Leu mutants expressed separately in Neuro-2a stable cell lines were characterized by an increase in the Thr286 phosphorylation, probably due to the disruption of the interaction between the kinase domain and the regulatory segment responsible for the autoinhibition of its kinase activity. Furthermore, transfecting primary hippocampal neurons with the Pro212Gln mutant also resulted in significantly increased A-type K^+^ currents. Another group reported on a patient with autism spectrum disorder carrying a Glu183Val *CAMKA2A* variant [1]. The variant decreased CaMKII substrate phosphorylation and the Thr286 autophosphorylation of both mutant and wild-type proteins, suggesting that the mutant acts in a negative-dominant manner. Furthermore, expression of the same variant in the hippocampal neurons altered the dendritic morphology (increases dendritic arborization and decreases dendritic spine density) and increased excitatory synaptic transmission. Mice with CaMKIIα-Glu183Val variant have reduced CaMKII protein levels and display abnormal behavior characterized by hyperactivity, social deficits, and increased repetitive behavior. Moreover, Kury et al. studied the mutations identified in their cohort both in vitro and in vivo, obtaining similar results showing that some mutations had increased and others decreased autophosphorylation at Thr286 [2]. In addition, all mutations affected neuronal migration, highlighting the importance of autophosphorylation in neurodevelopment.

In a recent report, adult expression of wild-type *CAMK2A* in an inducible *Camk2a* mouse model rescues the behavioral and electrophysiological phenotypes observed in the *Camk2a* knock-out mice [21]. These results are very important for future therapies in patients affected by *CAMK2A* intellectual disability syndrome, as they show that dysregulation of *CAMK2A* expression can be reverted in vitro.

In our study, we propose through a structural interpretation how the deletion of Lys292 may affect the function of the CAMK2A protein. The amino acid is within the calmodulin binding domain, a region also able to interact with α-actinin-2. The region is mainly folded as a helix, and the deletion of an amino acid within the helix alters its correct conformation. Consequently, the geometrical features needed for the correct interaction are probably lost. As shown in Figure 4, Lys292 has the correct orientation to form H-bonds’ interactions with an interacting protein. The deletion of this amino acid can determine a loss of H-bonds in protein–protein interactions related to the protein function. Moreover, Arg296 can form salt–bridge interaction, and in case of deletion of Lys292, the orientation of the Arg296 side chain may be modified, with consequent inability to correctly form the interaction.

We are aware that the present report lacks an in vitro or in vivo functional study though the variant can be classified as “likely pathogenetic”, as it satisfies several criteria set by the American College of Medical Genetics guidelines. Indeed, we plan to perform functional studies on this variant in the near future in order to add further evidence of pathogenicity.

In summary, this study has described a new variant associated with intellectual developmental disorder autosomal dominant 53 (OMIM#617798), a recently discovered syndrome. We think that newly identified variants such as ours should always be described in the literature in order to assist other laboratories/clinicians in the genetic diagnosis of other patients with the same variant. The recent introduction of NGS technology in the clinical setting has increased a lot the diagnostic yield of neurodevelopmental disorders and other rare diseases. Exome se-quencing results often consists of many variants (up to ten) which could be correlated with the phenotype. In this context it becomes very important to deeply characterize the patient from a clinical point of view and to collect as much as possible information concerning his personal and family history. In this regard, clinical cohort studies fo-cusing on a single novel gene and accurate clinical description of single cases represent a precious resource for the clinical geneticist during phenotype reevaluation as they can be of fundamental importance to reach a clinical diagnosis and make the final genotype–phenotype association. At this stage, with the updated literature resources, the clinical geneticist will select the variant (or variants) which mostly fits with the phenotype of the patient. Such an approach is known as “reverse phenotyping” and has been recently implemented in the clinical settings following the results emerging from exome se-quencing.

## Figures and Tables

**Table 1:** Genotype–phenotype correlation for the *CAMK2A* gene.

Patient	Variant	Type of Variant	ID	Seizure	Microcephaly	Language	Hypotonia	Affected Domain	Ref.
1	Pro212Gln	Missense	Severe	Yes	Yes	Not verbal	Yes	Kinase domain	[20]
2	Pro235Leu	Missense	Severe	Yes	No	Not verbal	Not specified	Kinase domain	[20]
3	c.817 − 1G > A	Splicing	Severe	Yes	No	Not specified	Yes	Regulatory domain	[20]
4	Gly22Glufs*10	Frameshift	Moderate	No	No	Delayed speech	No	Kinase domain	[2]
5	Phe98Ser	Missense	Moderate	No	No	Delayed speech	Yes	Kinase domain	[2]
6	Glu109Asp	Missense	Severe	Yes	No	Delayed speech	Yes	Kinase domain	[2]
7	Ala112Val	Missense	Severe	No	No	Delayed speech	Yes	Kinase domain	[2]
8	Glu183Val	Missense	Mild	No	No	Delayed speech	No	Kinase domain	[2]
9	c.598 + 2dup	Missense	Severe	No	No	Delayed speech	No	Not reported	[2]
10	Pro212Leu	Missense	Severe	Yes	No	Delayed speech	Yes	Kinase domain	[2]
11	Pro212Leu	Missense	Mild-severe	Yes	Yes	Delayed speech	Yes	Kinase domain	[2]
12	Pro212Leu	Missense	Moderate	No	No	Delayed speech	No	Kinase domain	[2]
13	Pro235Leu	Missense	Mild	No	No	Delayed speech	No	Kinase domain	[2]
14	c.817 − 1G > A	Splicing	Severe	Yes	No	Delayed speech	Yes	Not reported	[2]
15	His282Arg	Missense	Severe	No	No	Delayed speech	No	Regulatory domain	[2]
16	Thr286Pro	Missense	Severe	No	No	Delayed speech	Yes	Regulatory domain	[2]
17	c.1204 + 1G > A	Splicing	Mild	No	No	No	No	Not reported	[2]
18	c.874_876delCTT, p. Lys292del	Inframe deletion	Severe	Yes	Yes	Not verbal	Yes	Regulatory domain	Our patient

## Data Availability

Research data are not shared.
